# The Advantages and Disadvantages of Online and Blended Therapy: Survey Study Amongst Licensed Psychotherapists in Austria

**DOI:** 10.2196/11007

**Published:** 2018-12-18

**Authors:** Raphael Schuster, Raffaela Pokorny, Thomas Berger, Naira Topooco, Anton-Rupert Laireiter

**Affiliations:** 1 Outpatient Center for Clinical Psychology, Psychotherapy and Health Psychology Department of Psychology University of Salzburg Salzburg Austria; 2 Faculty of Psychology University of Vienna Vienna Austria; 3 Department of Clinical Psychology and Psychotherapy University of Berne Berne Switzerland; 4 Department of Behavioural Sciences and Learning Linköping University Linköping Sweden

**Keywords:** computer-assisted therapy, eHealth, psychotherapy, attitude of health personnel, attitude to health, mobile phone

## Abstract

**Background:**

Web-based and blended (face-to-face plus Web-based) interventions for mental health disorders are gaining significance. However, many licensed psychotherapists still have guarded attitudes toward computer-assisted therapy, hindering dissemination efforts.

**Objective:**

The objective of this study was to provide a therapist-oriented evaluation of Web-based and blended therapies and identify commonalities and differences in attitudes toward both formats. Furthermore, it aimed to test the impact of an information clip on expressed attitudes.

**Methods:**

In total, 95 Austrian psychotherapists were contacted and surveyed via their listed occupational email address. An 8-minute information video was shown to half of the therapists before 19 advantages and 13 disadvantages had to be rated on a 6-point Likert scale.

**Results:**

The sample resembled all assessed properties of Austrian psychotherapists (age, theoretical orientation, and region). Therapists did not hold a uniform overall preference. Instead, perceived advantages of both interventions were rated as neutral (*t*_94_=1.89, *P*=.06; *d*=0.11), whereas Web-based interventions were associated with more disadvantages and risks (*t*_94_=9.86, *P*<.001; *d*=0.81). The information clip did not excerpt any detectable effect on therapists’ attitudes (*r*_95_=−.109, *P*=.30). The application of modern technologies in the own therapeutic practice and cognitive behavioral orientation were positively related to the given ratings.

**Conclusions:**

This study is the first to directly compare therapists’ attitudes toward Web-based and blended therapies. Positive attitudes play a pivotal role in the dissemination of new technologies, but unexperienced therapists seem to lack knowledge on how to benefit from technology-aided treatments. To speed up implementation, these aspects need to be addressed in the development of new interventions. Furthermore, the preference of blended treatments over Web-based interventions seems to relate to avoidance of risks. Although this study is likely to represent therapists’ attitudes in countries with less advanced electronic health services, therapists’ attitudes in more advanced countries might present differently.

## Introduction

In recent years, the amount of research on Web-based interventions has increased exponentially, and evidence supporting the efficacy of guided Web-based interventions in treating common mental health disorders has grown substantially [1-3]. As a consequence, knowledge obtained about Web-based interventions is also being transferred into conventional psychotherapy.

Web-based interventions are usually regarded as self-guided or therapist-guided internet or mobile phone-based programs, following one or more predefined treatment paths and entailing a given number of modules or exercises to be completed [4]. Blended interventions, in turn, are integrated combinations of face-to-face therapy together with the above-mentioned Web-based or mobile phone-based programs. In blended treatment, the application of computer-supported elements is intended to optimize the therapeutic process [5,6] or increase treatment efficiency [7] or effectiveness [8,9]. Blended treatment can be provided in individual or group therapy settings [10,11].

Web-based interventions exhibit a variety of advantages, such as good accessibility, flexibility, and cost and time savings [12]. Patients can enter Web-based interventions anonymously wherever and whenever they wish, resulting in low barriers to treatment and the exploitation of new ways of treating mental health disorders [13]. With regard to achievable treatment effects, meta-analyses indicate good effectiveness for Web-based interventions [1-3]. Despite the promising results, internal and external factors seem to hinder the broad dissemination of ready-to-use programs [14]. Important internal challenges are restricted tailoring to patient needs, challenges with managing comorbidity and acute crisis [12], and low patient engagement and high dropout rates [15]. External challenges exist in the form of national legal restrictions (as in force in Austria or Germany) or stakeholders´ cautious attitudes toward Web-based therapy [16-18].

For blended therapy, the internal and external preconditions appear to be different. Given the nature of blended therapy, some of the inherent drawbacks of Web-based treatment seem to be less challenging (eg, handling of crisis or suicide risk). Additionally, external restrictions are either nonexistent (eg, national laws) or appear to be less critical (eg, stakeholder attitudes [18]). On the other hand, blended therapy is typically associated with drawbacks such as reduced scalability owing to the reliance on personal therapist time and concomitantly higher treatment costs. Furthermore, the current evidence base of blended therapy is less comprehensive compared with Web-based interventions [10]. On the patient’s side, risks concerning restricted time for personal interaction [11], a potentially weakened patient-therapist bonding, or difficulties in communicating less apparent aspects of disease-related problems are of interest [19].

Regarding therapists’ attitudes toward Web-based (and blended) therapy, most findings from previous studies have shown that therapist appraisals of *Web-based interventions* range from cautiously positive to generally positive [20-22]. One study (N=1532) found that therapists were more skeptical regarding Web-based therapy compared with addressed patients (η_p_^2^=0.38) [20]. Although partly inconsistent, several studies have identified associations between theoretical orientation (eg, psychodynamic vs others) and attitudes toward the use of Web-based interventions [20,22,23]. Furthermore, therapists’ personal experience with the use of computer and media was found to positively relate to given appraisals (preference for Web-based treatment: 17.5% vs 6.4%; N=1104) [24]. Additionally, perceived applicability seems to depend on the appraisal of specific treatment features; for example, Web-based interventions were considered better suited to treat mild to moderate disorders [24]. Equally, therapist-level barriers relate to perceived disadvantages of Web-based therapy. Concerns exist with regard to potential negative effects (eg, on the working alliance) or doubtful treatment efficiency [17,25]. Literature on therapists’ attitudes of *blended therapy*, however, is less extensive, and some studies have not fully differentiated between Web-based interventions and more blended forms of therapy [17,26]. Therapists frequently have reported benefits such as improvements in patients’ self-management skills, improved access to therapy materials and treatment transparency, less traveling time, and possible reductions in so-called therapist drift-offs [5,19]. In a survey on the acceptability of computer-assisted therapy (N=1067) [17], professionals reported they were likely to integrate computer-supported therapies into their practice, but some doubted that the use of technology would actually improve treatment outcomes (low performance expectancy). Attitudes were also related to the general openness to new treatments (beta=.35) and computer literacy (beta=.19), and therapists varied in their ability and willingness to use computer-assisted programs. In a Delphi study (N=21), lack of nonverbal communication and the unsuitability for all patients were identified as disadvantages, and some therapists were concerned about blended therapy being time consuming or hindering to the rapport of less clear disease aspects or the establishment of a therapeutic relation [19]. Literature from neighboring disciplines, such as therapy monitoring or virtual reality, reveals comparable findings [27,28].

Psychotherapists play multiple roles in the dissemination of technology-assisted treatments [29,30] and will be end users of blended therapy; for example, Web-based interventions can be prescribed as an initial, adjunctive, or maintenance program. At the same time, psychotherapists hold important occupational and political positions in mental health systems. Therefore, it is important to improve the understanding of therapists’ attitudes toward Web-based and blended therapies.

Important issues of therapists’ attitudes toward Web-based and blended therapies refer to different levels of detail. For a global picture, the overall appraisal of both treatment strategies is of interest; for example, do psychotherapists hold a uniform preference for blended therapy over Web-based therapy? At a deeper level, separate rankings and comparative profiles can depict specific advantages and disadvantages and can lead to a better understanding of each intervention’s assigned strengths and weaknesses; for example, which advantages of Web-based therapy do therapists value most? At the highest level of resolution, stakeholders and developers might be interested in specific aspects of both treatments. Here the study provides an item-level analysis of both treatments; for example, do therapists believe that blending can improve current therapy practices? As a last aspect, we were interested in whether a short information video would influence therapists’ appraisals.

## Methods

### Survey Development and Design

To explore the outlined issues, a survey study was conducted. All randomly selected subjects received an email invitation to participate in the study. The corresponding survey contained demographic information as well as items on perceived advantages and disadvantages of Web-based and blended therapies. Questions on both therapy forms were organized in separate blocks, which were presented in randomized order.

#### Demographic Information

Therapists reported on their educational and professional background, their years in profession (training period excluded), their basic professions (psychology, pedagogics, social work, etc), and their working region (urban vs rural). Additionally, we gathered descriptive information on self-reported computer and internet usage behavior and a ranking of blended therapy applications.

#### Construction of Survey Questions and Factor Analysis

Because there was no questionnaire designed to contrast the differences in the perceived (dis) advantages of Web-based and blended therapies among psychotherapists, we constructed a survey based on previous literature in the field. In the first step, an extensive literature search was conducted. In the next step, 4 previous studies with high relevance were identified [17,22,31,32] and served as the basis for this survey. In the last step, additional research was regarded during the construction of the items. The item selection was based on different criteria with a scope on 3 main categories (basic characteristics, therapeutic process, and health care perspective). Several items regarded the basic characteristics of Web-based and blended interventions (eg, treatment flexibility, age, or suitability). Further items were related to advantages and disadvantages for the therapeutic process and the therapeutic alliance (eg, repetition of therapy material, complexity of treatment, or nonverbal signals). The last category contained items assessing therapists’ attitudes about occupational interests, the psychotherapy supply, and the evidence base of both treatments (eg, treatment quality, data security, or health care provision). Finally, 32 items were selected from a total of 54 candidate items. Selection criteria were redundancy and relevancy of items as well as fit for both intervention types. The selection was carried out consensually by the first, second, and last author (RS, RP, and AL, respectively). A detailed assignment of each item’s theoretical background is provided in Multimedia Appendix 1.

Ratings were made on a 6-point Likert scale, and items were divided into “perceived advantages” and “perceived disadvantages” for each intervention strategy (6=I definitely agree, 5=I agree, 4=I somewhat agree and so on). With the exception of the respective intervention name, the items of both scales were identical. Both scales showed high internal consistency (18 items for advantages, Cronbach alpha=.931; 13 items for disadvantages, alpha=.930). Because factor analyses in small samples (100 individuals) can be applied, when the observed communalities were high (λ>0.6) [33], we conducted a maximum likelihood factor analysis (rotation based on the Varimax method) to roughly explore the basic factor structure of our questionnaire. The analysis revealed a single factor with high factor loadings (average λ=0.680). Here perceived advantages were related positively and disadvantages negatively to the identified factor. Detailed results of the factor analysis are listed in Multimedia Appendix 1.

#### Production of the Video Clip

An 8-minute video clip presented the definitions and the usual content of Web-based and blended interventions as well as their evidence base. There was no particular sequence on advantages and disadvantages of both interventions. The video clip consisted of a sequence of presentation slides depicting graphs, tables, and text-based information on unguided and guided Web-based interventions as well as on blended therapy. A professional rehearsal voice recorded the audio stream. The video did not feature any visible speaker or interview partner (eg, psychologist, professor, or patient).

### Procedure

Therapists were contacted via the national register for licensed psychotherapists administered by the Federal Ministry of Health and Women in Austria, renamed and reorganized into the Federal Ministry of Labour, Social Affairs, Health and Consumer Protection (Bundesministerium für Arbeit, Soziales, Gesundheit und Konsumentenschutz) after the 2017 state elections. The register contains a comprehensive list of all licensed psychotherapists in Austria (N=8643) and is frequently used for research purposes. The entire register was downloaded, and 12.14% (1050/8643) of the addresses were selected at random. Therapists were invited to participate in the survey via email, and the survey was provided via a Web-based survey platform (LimeSurvey). The cover letter was entitled *Survey on Web-based and blended interventions in psychotherapy* and entailed information on the study background, purpose, privacy issues, and detailed contact information. Following best practice guidelines (eg, Tailored Design Method [34]), efforts were made to keep the perceived costs of responding low (eg, easy to complete), to address the relevance (eg, currency of the topic) and the benefits of participating (eg, 3×20 Euro tombola), and to establish trust by ensuring data security and a professional presentation. Additionally, we attempted to provide therapists with basic knowledge about both interventions by screening an 8-minute video clip at the beginning of the survey. Owing to the conflicting priorities of providing some information on the topic but not interacting with personal attitudes, we decided to randomly present this video clip to 50% of the surveyed psychotherapists. Answering the survey took 23 minutes on average.

### Analyses

Statistical analyses were conducted using SPSS Statistics 23 (IBM SPSS Statistics). Responses were based on mandatory field completion; thus, no missing data arose. Results were not normally distributed and nonparametrical statistics therefore would be indicated. For reasons of interpretability, we preferred to present the investigated differences in terms of Cohen *d* [35]. Therefore, obtained data were analyzed parametrically (*t* tests) and nonparametrically (Wilcoxon tests). Because the results corresponded almost perfectly, we decided to present the results based on *t* tests together with effect sizes in Cohen *d*. Differences in demographic variables between surveyed therapists and the population of Austrian therapists were analyzed using chi-square tests. Influences of demographic variables (eg, occupational computer usage or therapeutic orientation) and the impact of the presented video clip were analyzed using point-biserial correlations.

Dependent sample item-level *t* tests were applied to contrast both interventions against each other (19 positive items and 13 negative items). We decided to adjust for type l error inflation by applying Bonferroni correction to each scale separately, resulting in a critical *t* value of *t*=2.78 for advantages and *t*=2.65 for disadvantages. Results below the critical threshold are labeled in the corresponding tables. Here the average value of a given item (eg, treatment flexibility) was tested against the total value of the corresponding subscale (eg, advantages of Web-based interventions). According to power analyses (G*Power 3 [36]), the calculated power to detect a given effect size of *d*=0.3 and *d*=0.5 was beta=.83 and beta=.99, respectively.

## Results

### Surveyed Therapists

In response to our nationwide invitation, 95 out of 1050 contacted therapists completed the survey between May 2016 and June 2016, resulting in a response rate of 9.31%. The information clip was presented to 48% (46/95) therapists. For estimating representativeness and potential selection biases, information on therapists’ theoretical orientation (eg, cognitive behavioral therapy) and other features are provided in Tables 1 and 2. Among the surveyed psychotherapists, 65% (62/95) were female, which corresponded to the population of psychotherapists in Austria (71.8%). The proportion of behavioral psychotherapists in Austria (11.9%) is traditionally lower than that in other German-speaking countries, such as Germany (35%) [37]. This was reflected in our sample (14/95, 15%). Apart from humanistic therapists, our sample seems to largely reassemble the population of Austrian therapists. Another important feature of our sample is the full range of possible professions a licensed psychotherapist in Austria may originate from. Only 44% (42/95) of the surveyed therapists were psychologists. The remaining 56% (53/95) stemmed from diverse professional areas, such as medicine, social work, etc. Survey results can benefit from this heterogeneity because many different perspectives entered the appraisal of Web-based and blended therapies.

### Therapists’ Computer and Internet Behavior

The vast majority of our sample used computers regularly for email correspondence and for Web-based search (Table 3). Regular email contact with clients was substantially lower, and only 12%-13% already used computers for videoconferencing or to supply modern media, videos, or book chapters to patients.

**Table 1:** Demographic characteristics of the sample.

Characteristics		Sample (N=95)	Population of psychotherapists in Austria (N=8643)		Statistics		
					χ² value		*P* value
**Gender, n (%)**							
	Female	62 (65.26)	6205 (71.79)		2.1		.14
	Male	33 (34.73)	2438 (28.21)		2.1		.14
Age in years, mean (SD)		48.7 (12.2)	N/A^a^		N/A		N/A
**Theoretical orientation, n (%)**							
	Psychodynamic or analytic	28 (29.47)	2230 (25.80)		0.6		.44
	Humanistic	26 (27.36)	3232 (37.39)		4.0		.04
	Behavioral	14 (14.73)	1029 (11.90)		0.7		.39
	Systemic	27 (28.42)	2152 (24.90)		0.6		.44
Region (urban/rural), %		76.8/23.2	70/30		2.1		.15

^a^N/A: not applicable.

**Table 2:** Professional characteristics of the sample.

Characteristics		Value
**Basic profession, n (%)**		
	Psychology	42 (44)
	Counseling	7 (7)
	Medicine	5 (5)
	Social work	6 (6)
	Education	6 (6)
	Pedagogics	6 (6)
	University professor	2 (2)
	Theology or philosophy	3 (3)
	Nursing	2 (2)
	Economy or management	4 (4)
	Other	4 (4)
	No specification	8 (8)
Years in profession, mean (SD)		12.4 (11.3)

**Table 3:** Therapists’ occupational computer usage data (N=95).

Computer usage	Yes, n (%)	No, n (%)
General computer use (daily)	93 (98)	2 (2)
General email use (daily)	91 (96)	4 (4)
Conduct Web-based search	83 (87)	12 (13)
General administration tasks	77 (81)	18 (19)
Patient related documentation tasks^a^	41 (43)	54 (57)
Daily patient contact (email)^a^	45 (47)	50 (53)
Application of modern media during therapy^a^	13 (14)	82 (86)
Use of video conferencing^a^	12 (13)	83 (87)

^a^Activities that are relevant to Web-based and blended therapies.

### Overall Differences in Perceived Advantages and Disadvantages

The primary aim of this survey was to depict advantages and disadvantages of each intervention strategy at the item level. Still, the overall perception of each method’s (dis)advantages helps to reveal general attitudes. With scores of mean values of 3.45 and 3.61, the rating of perceived advantages can best be described as neutral (3=“I somewhat disagree;” 4=“I somewhat agree”). Although average perceived advantages of blended and Web-based interventions only differed tentatively with a small effect (*t*_94_=1.89, *P*=.06; *d*=0.11), the appraisal of possible disadvantages differed strongly with a high effect to the detriment of Web-based interventions (*t*_94_=9.86, *P*=.01; *d*=0.81).

### Rankings of Advantages and Disadvantages

Tables 4, 5, 6, and 7 present rankings of the most important advantages and disadvantages separated for each intervention. For each table, the average deviation from the scale mean was calculated. Perceived advantages of Web-based interventions (Table 4) on average scored mean of 3.45 (SD 0.72). With an average of mean of 3.61 (SD 0.58), blended interventions scored slightly above this value (4=“I somewhat agree”). Perceived disadvantages of Web-based interventions (Table 6) on average scored mean of 4.24 (SD 0.59). With an average of mean of 3.66 (SD 0.45), blended interventions scored significantly below this value.

### Comparison Between Both Interventions

This section analyzes the most salient differences between both interventions. Table 8 presents differences in perceived advantages between Web-based and blended interventions. Besides absolute deviations of both scores, effect sizes of the deviations are also provided as a standardized indicator. Table 9 presents differences in perceived disadvantages between both interventions.

**Table 4:** Ranking of advantages of Web-based interventions, deviation from average (N=95).

Rank number	Advantage	Score	
1	Bridging distances	4.80^a^	
2	Discrete	4.36^a^	
3	Timewise flexible	4.35^a^	
4	Psychoeducation	3.97^b^	
5	Repetition of work material	3.97^b^	
6	Suitable for young patients	3.92^b^	
7	Helping minorities or underserved	3.77^c^	
8	Contemporary	3.76^c^	
9	Bridging waiting time	3.71^c^	
10	Low threshold to care	3.58	
11	Web-based disinhibition effect	3.41	
12	Suitable for people with age >50	3.28	
13	Improve self-management	3.13^c^	
14	Delivering evidence-based treatment	2.99^b^	
15	Easy to share with family	2.93^b^	
16	Improvement of treatment quality	2.47^a^	
17	Can support therapist	2.45^a^	
18	Independency from therapist	2.40^a^	
19	Treatment intensification	2.33^a^	
Average	N/A^d^		3.45

^a^*P*<.001 of deviation from average.

^b^*P*<.01 deviation from average.

^c^*P*<.05 deviation from average.

^d^N/A: not applicable.

**Table 5:** Ranking of advantages of blended interventions, deviation from average (N=95).

Rank number	Advantage	Score
1	Bridging distances	4.47^a^
2	Discrete	4.45^a^
3	Psychoeducation	4.21^b^
4	Contemporary	4.03^b^
5	Bridging waiting time	4.03^b^
6	Helping minorities or underserved	3.97^b^
7	Repetition of work material	3.95^b^
8	Suitable for young patients	3.91^c^
9	Low threshold to care	3.87^c^
10	Timewise flexible	3.75
11	Suitable for people with age >50	3.63
12	Treatment intensification	3.43
13	Improvement of treatment quality	3.38^c^
14	Delivery of evidence-based treatment	3.24^c^
15	Improve self-management	3.22^b^
16	Web-based disinhibition effect	3.04^b^
17	Easy to share with family	2.85^a^
18	Can support therapist	2.72^a^
19	Independency from therapist	2.38^a^
Average	N/A^d^	3.61

^a^*P*<.001 deviation from average.

^b^*P*<.01 deviation from average.

^c^*P*<.05 deviation from average.

^d^N/A: not applicable.

**Table 6:** Ranking of disadvantages of Web-based interventions, deviation from average (N=95).

Rank number	Disadvantage		Score
1	Lack of nonverbal signals		5.11^a^
2	Missing important disease aspects		4.87^a^
3	Missing problems in therapeutic process		4.83^a^
4	Not applicable for the majority		4.66^b^
5	Data security issues		4.57^c^
6	Avoidance of difficult situation		4.49^c^
7	Risk of therapy discontinuation		4.22
8	Dealing with crisis		4.18
9	Too much technology		4.03^c^
10	Might result in side effects		3.89^c^
11	Transfer into daily life		3.66^c^
12	Technology devaluates therapist’s work		3.53^c^
13	More complicated than classical therapy		3.08^c^
Average	N/A^d^		4.24	

^a^*P*<.001.

^b^*P*<.01.

^c^*P*<.05.

^d^N/A: not applicable.

**Table 7:** Ranking of disadvantages of blended interventions, deviation from average (N=95).

Rank number	Disadvantage	Score
1	Data security issues	4.4^a^
2	Lack of nonverbal signals	4.08^b^
3	Not applicable for the majority	4.02^c^
4	Missing problems in therapeutic Process	3.87^c^
5	Missing important disease aspects	3.85^c^
6	More effortful than classical therapy	3.78
7	Avoidance of difficult situation	3.77
8	Might result in side effects	3.77
9	Risk of therapy discontinuation	3.57
10	Transfer into daily life	3.43^c^
11	Too much technology	3.32^c^
12	Technology devaluates therapist’s work	3.02^a^
13	Dealing with crisis	2.74^a^
Average	N/A^d^	3.66

^a^*P*<.001 deviation from average.

^b^*P*<.01 deviation from average.

^c^*P*<.05 deviation from average.

^d^N/A: not applicable.

**Table 8:** Comparison of advantages between Web-based and blended interventions (independent *t* tests; N=95).

Advantages	Blended interventions	Web-based interventions	Mean (SD)	Mean Cohen *d*
Treatment intensification	3.43	2.33	1.11^a^ (1.14)	0.97
Improvement of treatment quality	3.38	2.47	0.91^a^ (1.17)	0.77
Suitable for people with age >50 years	3.63	3.28	0.35^a^ (0.78)	0.45
Bridging waiting time	4.03	3.71	0.32^b^ (1.17)	0.27
Low threshold care	3.87	3.58	0.29^c^ (1.18)	0.25
Contemporary	4.03	3.76	0.27^b^ (0.86)	0.31
Can support the therapist	2.72	2.45	0.27^b^ (0.93)	0.29
Delivering evidence-based treatments	3.24	2.99	0.25^b^ (1.01)	0.25
Psychoeducation	4.21	3.97	0.24^c^ (0.97)	0.25
Helping minorities or underserved	3.97	3.77	0.20 (1.06)	0.19
Improve self-management	3.22	3.13	0.09 (0.77)	0.12
Discrete	4.45	4.36	0.09 (1.27)	0.07
Suitable for young patients	3.91	3.92	−0.01 (1.05)	−0.01
Independency from therapist	2.38	2.40	−0.02 (1.19)	−0.02
Repetition of work material	3.95	3.97	−0.02 (0.85)	−0.02
Easy to share with family	2.85	2.93	−0.08 (1.02)	−0.08
Bridging distances	4.47	4.80	−0.33^b^ (1.05)	−0.31
Web-based disinhibition	3.04	3.41	−0.37^b^ (1.30)	−0.28
Timewise flexible	3.75	4.35	−0.60^c^ (1.51)	−0.40

^a^*P*<.001.

^b^*P*<.01.

^c^*P*<.05.

### Additional Findings

Additionally, we investigated the relation between demographic variables as well as the 2 variants of therapists’ occupational computer usage (wide and narrow perspective) and therapist attitudes. Age (*r*_95_=−.019, *P*=.85), years in profession (*r*_95_=−.062, *P*=.55), gender (*r*_95_=.039, *P*=.71), rural workplace (*r*_95_=−.060, *P*=.57), or presentation of the short video clip (*r*_95_=−.109, *P*=.30) did not relate to given appraisals but computer usage did. In the wide perspective of therapists’ occupational computer usage (all 4 marked variables from Table 3), a trend toward more favorable attitudes was found (*r*_95_=.177, *P*=.09). In the narrow perspective (application of modern media or videoconferencing; the last 2 items presented in Table 3), this relation became more evident (*r*_95_=.241, *P*=.02). Finally, we correlated the therapeutic orientation with attitudes, and found a trend toward more positive attitudes among behavioral therapists (*r*_95_=.188, *P*=.07). As the last aspect, we were interested in the perceived applicability of blended therapy elements as well as in therapists’ interest in potentially applying such elements. Corresponding results are listed in Tables 10 and 11.

**Table 9:** Comparison of disadvantages between Web-based and blended interventions (independent t tests; N=95)*.*

Disadvantages	Web-based interventions	Blended interventions	Mean (SD)	Mean Cohen *d*
Dealing with crisis	4.18	2.74	1.44^a^ (1.17)	1.22
Lack of nonverbal signals	5.11	4.08	1.03^a^ (1.16)	0.89
Missing important disease aspects	4.87	3.85	1.02^a^ (1.14)	0.89
Missing problems in therapeutic process	4.83	3.87	0.96^a^ (1.02)	0.94
Avoidance of difficult situation	4.49	3.77	0.72^a^ (1.25)	0.58
Too technological	4.03	3.32	0.71^a^ (1.38)	0.51
Risk of therapy discontinuation	4.22	3.57	0.65^a^ (1.16)	0.56
Not applicable for the majority	4.66	4.02	0.64^a^ (1.31)	0.49
Technology devaluates therapist’s work	3.53	3.02	0.51^a^ (1.39)	0.37
Transfer into daily life	3.66	3.43	0.23^b^ (1.06)	0.22
Data issues	4.57	4.40	0.17 (1.13)	0.15
Might result in side effects	3.89	3.77	0.12 (1.11)	0.11
More effortful than classical therapy	3.08	3.78	−0.70^a^ (1.24)	−0.56

^b^*P*<.05.

^a^*P*<.001.

**Table 10:** Applicability of blended therapy elements (N=95).

Applicability of elements	%
Psychoeducation	96
Record about mood and activities	85
Web-based diary	84
Exercises at home (homework)	84
Videos and multimedia (like YouTube)	78
Mediation and relaxation exercises	74
Diary on smartphone	63
Reflection of therapy elements	59
Introduction into treatment	52
Debriefing of the session	32

**Table 11:** Interest in blended therapy elements (N=95).

Interest in elements	%
Videos and multimedia (psychoeducation, short videos)	54
Communication (short message service text message, email, feedback about exercises)	45
E-learning (short texts, case example, Web-based exercises)	41
Smartphone or app (diary, behavioral observation, real-time-monitoring)	34
None of the components	26

## Discussion

### Principal Findings

This study contributes to the understanding of licensed psychotherapists’ attitudes toward Web-based and blended therapies. By focusing on different levels of detail (general appraisal, internal and comparative profiles, and item-level analyses), the perceived advantages and disadvantages of both interventions are depicted. Major findings concern the neutral perception of both interventions’ advantages as well as the increased perception of disadvantages of Web-based interventions. Additionally, a mismatch between therapists’ concepts about both interventions and the corresponding empiric evidence can be identified at the item level of analysis. Finally, the effect of an 8-minute information video was found to be negligible.

Therapists’ overall perception of advantages in Web-based and blended therapies can be described as neutral because average ratings ranged around the midpoint of the survey’s scale. Although this finding does not suggest negative attitudes toward both interventions, it seems to be more in line with studies suggesting that psychotherapists are reserved and cautious in their views [20,28,29]. Even though therapists might be expected to have more positive attitudes toward technology-aided, face-to-face therapy (blended format), there was no overall preference nor was there significant difference between both intervention formats’ advantages. Blended therapy pursues the frequently stated goal of unifying the advantages of traditional face-to-face and computer-supported treatments [10], and our results suggest that this relates primarily to risk-related aspects. According to the Diffusion Of Innovations theory [38] and the Unified Theory of Acceptance and Use of Technology [39], perceived advantages as well as compatibility with personal beliefs and preferences play a pivotal role in the successful dissemination of new technologies. Accordingly, the lack of perceived advantages or benefits can result in reduced interest and consequently, in the possible obstruction of dissemination efforts. In this context, several studies [17,24,40] have stressed the relevance of electronic health (eHealth) knowledge and experience as facilitators of more positive attitudes. We found a tendency toward more positive attitudes among therapists who had already used some computer or media support in their practice.

However, when comparing perceived disadvantages, the results were a bit different. Although the attributed disadvantages of blended therapy again can be described as neutral, the surveyed professionals showed particularly more negative attitudes toward the presented risks associated with Web-based interventions. This finding is in accordance with results from previous studies [20,41,42]. Some authors have ascribed the low acceptance of Web-based interventions to professionals’ concerns that their work may be replaced by such technologies [12,43]. Although surveyed therapists did not per se agree with the statement that technology would devaluate a therapist’s work (a minor disadvantage in Table 6), more negative attitudes toward Web-based interventions emerged when the approach was compared with blended treatment (Table 9). Still, the most salient disadvantages of Web-based therapy concerned therapeutic process aspects, such as the lack of nonverbal signals, missing important disease aspects, or dealing with crisis. Given that the relevance of these aspects differs between guided and unguided forms of Web-based therapy, further differentiation between both forms would have been advisable. In this regard, both guided and unguided forms of Web-based therapy are represented equally in this study. In the synopsis, both formats failed to elicit positive responses among psychotherapists. Additionally, risks and disadvantages seem to be particulary relevant to Web-based interventions, resulting in a more negative perception of this format. This result is in line with previous studies that reported on stakeholders’ and therapists’ overall preferences of blended therapy over Web-based interventions that are completely delivered via the internet [18,44] and suggests that perceived risks could play a pivotal role in this regard.

At the item level, a mismatch between empirical evidence and therapists’ personal beliefs was found. Recognizing such differences can help improve training and consumer information and thus improve the dissemination of internet-based interventions; for example, the empirical base of Web-based interventions in delivering evidence-based treatments was not acknowledged by surveyed professionals (Table 4 Rank 14). The same applies to the improvement in patients’ self-management abilities in blended therapy approaches—a benefit suggested in previous literature [6,19]. Finally, the increased salience of potential risks of Web-based interventions is currently not supported by evidence [45-47]. In this context, previous studies have successfully promoted positive attitudes toward eHealth in general and patient populations by providing text or video-based information [48,49]. At the same time, comparable studies yielded less successful results [50,51]. In this context, the mode of presentation (text vs video-based) and the use of persuasive methods (eg, expert evaluations or testimonials) [52] could influence the impact of the presented material. Whether such a strategy could change therapists’ attitudes toward Web-based or blended treatments for now remains an open question. In this study, the randomized presentation of a short information clip did not effect therapists’ attitudes. Ultimately, more profound implementation strategies appear most promising [53]. Among others, such strategies should focus on teaching, therapist trainings, incentives, and reimbursement policies.

In the light of the above-mentioned innovation theories (Diffusion Of Innovations theory and Unified Theory of Acceptance and Use of Technology [38,39]), a further strategy to improve uptake emphasizes on therapist-oriented co-design. On one hand, practitioners agreed that blended interventions constitute a contemporary and flexible approach. Simultaneously, a strikingly high number of therapists doubted that blended therapy would support them in their daily work (Table 4, rank 18 of 19). Thus, the criterion of *performance expectancy—* which was an identified key factor for (patient-based) acceptance and use in previous Web-based therapy studies [21,40]—remains unsatisfied from the therapists’ perspective on blended therapy. Furthermore, therapist-based *effort expectancy* for blended therapy is very high (Table 9, last item) because therapists do expect more workload from using blended formats. Although therapists frequently participate in the development of new interventions [54], developers should particularly emphasize how therapist-based performance and effort expectancies can be addressed in blended therapy.

Therapist attitudes were related to their personal experience in using modern technologies but not to work experience (years in profession) or other demographic variables. The relevance of personal experience has frequently been stressed in previous qualitative and quantitative studies [17,22-24,44]. Concerning the role of therapeutic orientation, our results revealed only a statistical tendency toward more positive attitudes among cognitive behavioral therapists. Thus, although our results contradict findings from several studies showing more negative attitudes among psychodynamic and humanistic therapists [20,23], they support studies identifying more liberal attitudes among behavioral therapists [22]. When interpreting these results, the small sample size, which further spreads over several different therapeutic orientations, needs to be taken into account.

Regarding the study’s validity, certain factors support representativeness, whereas others restrict generalizability. Essential features of the sample resemble available population characteristics (therapeutic orientation, gender, or regionality), suggesting that the attitudes of the respective sample represent those of Austrian therapists. However, recent literature indicates critical regional differences in the knowledge about and acceptability of internet-assisted and blended interventions. Stakeholders in countries with more advanced eHealth services tend to have more positive attitudes [18] and as previously mentioned, personal experience with technology- and media-supported therapy elements relates to more positive evaluations of both treatment formats [24]. Consequently, this study seems to primarily represent therapist attitudes in surroundings with less advanced eHealth services, whereas therapists in advanced eHealth environments might hold more positive attitudes.

### Limitations

This study has several limitations. First, considering that the study was carried out online and therapists were only contacted via email, selection bias may have been introduced. To counteract this tendency, it would have been advisable to use an additional paper-pencil version of the questionnaire [33]. Second, the low response rate increased the risk of introducing response bias. To estimate this risk, available population data on essential sample characteristics are provided, and corresponding deviations from the population range from around 3.1 to 10.4 percentage points. Although the sample can be considered representative for psychodynamic, behavioral, and systemic therapists (deviation=3.1%-3.2%), therapists with a humanistic orientation were underrepresented (deviation=10.4%). Third, the sample size in this study was rather small. Consequently, the study lacks sufficient power to detect small effects or subgroup effects reliably. Therefore, findings on the influence of therapeutic orientation or the relevance of personal experience in therapists’ appraisals should be interpreted with caution. Fourth, many previous studies have employed standardized questionnaires [31,32]. Owing to the specific aim of this study and the lack of a corresponding pretested questionnaire, we have not been able to implement any validated survey. As a result, the translation of the survey is prone to language errors, and assumptions about its factor structure are unconfirmed. However, the reported exploratory factor analysis does indicate a single factor structure in which factor loadings of advantages and disadvantages load according to expectations. Additionally, the full translation of each survey question is provided in Multimedia Appendix 1. As the last aspect, we assessed therapists’ daily personal computer usage, but we did not assess computer literacy by means of a standardized questionnaire. Applying computer literacy questionnaires might have led to additional findings.

### Conclusion

This study is the first to investigate therapist attitudes toward blended therapy and to directly compare therapist appraisals of Web-based and blended intervention formats. Therapists’ general attitudes can be described as neutral to cautious, and therapists’ preferences of blended therapy over Web-based interventions seem to be risk-driven. According to two mentioned innovation theories, positive beliefs and preferences play a pivotal role in the successful dissemination of new technologies. As one crucial aspect, therapists seem to lack knowledge on how to benefit from technology-aided treatments. This aspect should be regarded in the development of new interventions. However, contrary to personal experience with technology- and media-supported therapy, an unspecific information video did not influence therapists’ appraisals. In this context, the study provides a starting point for improved therapist education (eg, fostering knowledge on potential benefits or addressing frequent mismatches between empirical evidence and therapists’ concepts). Although this study is likely to represent therapist opinions in countries with less advanced eHealth services, the small sample size restricts its sensitivity to detect small or subgroup effects.

## Appendix

Multimedia Appendix 1 Full translation of the questionnaire and factor loadings.

## Abbreviations

eHealth: electronic health
