# Supplementary material for: The Advantages and Disadvantages of Online and Blended Therapy: Survey Study Amongst Licensed Psychotherapists in Austria
Source: J Med Internet Res. 2018 Dec 18;20(12):e11007. doi: 10.2196/11007 (PMC6315274; doi:10.2196/11007)
Supplement: Multimedia Appendix 1 [file jmir_v20i12e11007_app1.pdf]

## Appendix A. Full translation of the questionnaire and literature

Main literature sources for item construction:

Becker E, Jensen-Doss A: Computer-assisted therapies: Examination of therapist-level barriers to their use. Behav Ther 2013;44(4):614-624. DOI:10.1016/j.beth.2013.05.002.

Vigerland S, Ljótsson B, Bergdahl Gustafsson F et al.: Attitudes towards the use of computerized cognitive behavior therapy (cCBT) with children and adolescents: A survey among Swedish mental health professionals. Internet Interv 2014;1(3):111-117. DOI:10.1016/j.invent.2014.06.002.

Moritz S, Schröder J, Meyer B, Hauschildt M: The more it is needed, the less it is wanted: Attitudes toward face-to-face intervention among depressed patients undergoing online treatment. Depress Anxiety 2012;30:157-167. DOI:10.1002/da.21988.

Schröder J, Sautier L, Kriston L et al.: Development of a questionnaire measuring attitudes towards psychological online interventions–the APOI. J Affect Disord 2015;187:136-141. DOI:10.1016/j.jad.2015.08.044.

Full translation and additional literature sources:

| # | Advantage | Additional literature source |
|---|---|---|
| 1 | Online interventions make it easier to bridge geographical distances | [ 1 ] |
| 2 | With online interventions, patients need to fear less that others will find out about their therapy | [ 2 ] |
| 3 | Online interventions offer temporal flexibility | [ 1, 3 ] |
| 4 | With online interventions, psychoeducation about causes of diseases, treatment strategies or medication could be realized very well | [ 1, 4 ] |
| 5 | With online interventions, work material can be viewed repeatedly and worked on at different times | [ 2, 5 ] |
| 6 | Online interventions are especially interesting for younger generations | [ 27 ] |
| 7 | With online interventions, underserved groups could also be addressed | [ 5, 6 ] |
| 8 | Online interventions are contemporary | [ 5 ] |
| 9 | With online interventions, waiting times can be bridged due to fast availability and accessibility | [ 1, 5, 7 ] |
| 10 | With online interventions, low threshold care could be well implemented | [ 1, 5, 8, 9 ] |
| 11 | The anonymity with online interventions could lead to greater openness and sincerity (online disinhibition effect) | [ 1, 5, 10, 11, 12 ] |
| 12 | Online interventions are also suitable for the generation 50+ | [ 13 ] |
| 13 | With online interventions, self-efficacy is strengthened, because patients can work on the exercises and define the pace by themselves | [ 5, 13, 14) |
| 14 | Online interventions provide the possibility to implement evidence based treatment strategies | [ 1 ] |
| 15 | It could be easier for patients to speak to friends and family about the utilization of online interventions compared to classic therapy | [ 15 ] |
| 16 | Online interventions could improve the quality of treatment | [ 1, 16 ] |
| 17 | Online interventions can support (unexperienced) therapists | [ 5 ] |

| | | |
|---|---|---|
| 18 | With online interventions, patients are less dependent on the professional competence of their therapist | [ 16, 17 ] |
| 19 | Online interventions lead to a treatment intensification | [ 5 ] |
| **#** | **Disadvantage** | |
| 1 | With online interventions, there is a suboptimal exchange between therapists and patients due to a lack of non-verbal signals | [ 5, 10 ] |
| 2 | With online interventions, it is easier to overlook important disease aspects | [ 5, 17 ] |
| 3 | With online interventions, problems in the therapy process might be overlooked | [ 17 ] |
| 4 | Online interventions are not suitable for the majority of people with mental health problems | [ 18 ] |
| 5 | Online interventions hold the risk of data security issues | [ 5, 19, 20 ] |
| 6 | With online interventions, difficult topics are easier to avoid | [ 5, 13, 21 ] |
| 7 | Online interventions hold a greater risk of therapy discontinuation compared to classic therapy | [ 1, 22, 23 ] |
| 8 | With online interventions, there is no therapist present during a crisis | [ 1, 5, 17 ] |
| 9 | Online interventions are dehumanized and too technical | [ 24 ] |
| 10 | Online interventions hold the risk of unwanted side effects | [ 25 ] |
| 11 | It is probably harder to transfer suggestions of online interventions into daily life | [ 2 ] |
| 12 | Online interventions devalue the work of therapists | [ 26 ] |
| 13 | Online interventions seem to be more complicated than classic therapy | [ 4 ] |


1) Klein J, Berger T: Internetbasierte psychologische Behandlung bei Depressionen. Verhaltenstherapie 2013;23(3):149-159. DOI:10.1159/000354046.
2) Moritz S, Schröder J, Meyer B, Hauschildt M: The more it is needed, the less it is wanted: Attitudes toward face-to-face intervention among depressed patients undergoing online treatment. Depress Anxiety 2012;30:157-167. DOI:10.1002/da.21988.
3) Barak A, Hen L, Boniel-Nissim M, Shapira N: A Comprehensive Review and a Meta-Analysis of the Effectiveness of Internet-Based Psychotherapeutic Interventions. J Technol Hum Serv 2008;26(2-4):109-160. DOI:10.1080/15228830802094429.
4) van der Vaart R, Witting M, Riper H, Kooistra L, Bohlmeijer E, van Gemert-Pijnen L: Blending online therapy into regular face-to-face therapy for depression: content, ratio and preconditions according to patients and therapists using a Delphi study. BMC Psychiatry 2014;14(1):355. DOI:10.1186/s12888-014-0355-z.
5) Berger T, Andersson G: Internetbasierte Psychotherapien: Besonderheiten und empirische Evidenz. PPmP - Psychotherapie · Psychosomatik · Medizinische Psychologie 2009;59(03/04):159-170. DOI:10.1055/s-0028-1090162.
6) Preschl B, Maercker A, Wagner B: The working alliance in a randomized controlled trial comparing online with face-to-face cognitive-behavioral therapy for depression. BMC psychiatry 2011; 11(1): 189. DOI:10.1186/1471-244X-11-189

7) Andersson G, Cuijpers P: Internet-Based and Other Computerized Psychological Treatments for Adult Depression: A Meta-Analysis. Cogn Behav Ther 2009;38(4):196-205. DOI:10.1080/16506070903318960.
8) Ebert DD, Erbe D: Internetbasierte psychologische Interventionen; in M. Berking M, Rief W (ed): Klinische Psychologie und Psychotherapie für Bachelor. Heidelberg, Springer Medizin Verlag, 2012, pp 131-139. DOI:10.1007/979/-3-642-25523-6
9) Knatz, B. Rat und Hilfe aus dem Internet – Die Beratung per Mail. Standards und Herausforderungen. http://www.eberatungsjournal.net/ausgabe_0105/knatz.pdf January 2005. Assessed July 08 2016
10) Klasen M, Knaevelsrud C, Böttche M: Die therapeutische Beziehung in internetbasierten Therapieverfahren. Nervenarzt 2013;84(7):823-831. DOI:10.1007/s00115-012-3659-6.
11) Schultze, N. G. (2007). Erfolgsfaktoren des virtuellen Settings in der psychologischen Internet-Beratung. CyberPsy & Behav 2005;8(2):172-177. DOI:10.1089/cpb.2005.8.172.
12) Apolinário-Hagen J, Tasseit S: Chancen und Risiken der Internettherapie für die Regelversorgung in Deutschland. Ein Beitrag zur Psychotherapie im Zeitalter von Web 2.0. Fachzeitschrift für Onlineberatung und computervermittelte Kommunikation 2016;1(7): 69-86.
13) Caspar F, Berger T, Lotz-Rambaldi W, Hohagen F: Internetbasierte Psychotherapie und E-Mental-Health. Verhaltenstherapie 2013;23(3):137-139. DOI:10.1159/000355118.
14) Moritz S, Schilling L, Hauschildt M, Schröder J, Treszl A. A randomized controlled trial of internet-based therapy in depression. Behav Res Ther 2012;50(7-8):513-521. DOI:10.1016/j.brat.2012.04.006.
15) Andersson G, Titov N: Advantages and limitations of Internet-based interventions for common mental disorders. World Psychiatry. 2014;13(1):4-11. doi:10.1002/wps.20083.
16) Berger T, Caspar F. Internetbasierte Psychotherapien. Psychiatrie und Psychotherapie up2date 2011;5(01):29-43. DOI:10.1055/s-0030-1265934.
17) Newman M, Szkodny L, Llera S, Przeworski A: A review of technology-assisted self-help and minimal contact therapies for anxiety and depression: Is human contact necessary for therapeutic efficacy?. Clin Psychol Rev 2011;31(1):89-103. DOI:10.1016/j.cpr.2010.09.008.
18) Bauer S, Kordy H (ed): E-Mental-Health. Neue Medien in der psychosozialen Versorgung. Heidelberg, Springer, 2008
19) Yuen E, Goetter E, Herbert J, Forman E: Challenges and opportunities in internet-mediated telemental health. Professional Psychology: Research and Practice 2012;43(1):1-8. DOI:10.1037/a0025524.
20) Kersting A, Schlicht S, Kroker K: Internettherapie. Nervenarzt 2009;80(7):797-804. DOI:10.1007/s00115-009-2721-5.
21) Caspar F: Technological developments and applications in clinical psychology and https://online.uni-salzburg.at/plus_online/webnav.inipsychotherapy: Introduction. J Clin Psychol 2004;60(3):221-238. DOI:10.1002/jclp.10260.
22) Spek V, Cuijpers P, Nyklicek I, Riper H, Keyzer J, Pop V: Internet-based cognitive behaviour therapy for symptoms of depression and

anxiety: a meta-analysis. Psychol Med 2007;37(03):319. DOI:10.1017/s0033291706008944.
23) Schröder J: Psychometrische Messung von Einstellungen gegenüber psychologischen
Online-Interventionen bei depressiven Personen und Psychotherapeuten. (Dissertation, Leipzig, Deutsche Nationalbibliothek). http://d-nb.info/1074642678/34
October 2015. Assessd July 10, 2016
24) Stetina, BU, Kryspin-Exner I (ed): Gesundheit und Neue Medien. Psychologische Aspekte der Interaktion mit Informations- und Kommunikationstechnologien. Wien, Springer Verlag, 2009.
25) Waller R, Gilbody S: Barriers to the uptake of computerized cognitive behavioural therapy: a systematic review of the quantitative and qualitative evidence. Psychol Med 2008;39(5):705-712. DOI:10.1017/s0033291708004224.
26) Kenter R, van de Ven P, Cuijpers P et al.: Costs and effects of Internet cognitive behavioral treatment blended with face-to-face treatment: Results from a naturalistic study. Internet Interv 2015;2(1):77-83. DOI:10.1016/j.invent.2015.01.001.
27) Ellis L A, Collin P, Hurley P J, Davenport T A, Burns J M, Hickie I B: Young men’s attitudes and behaviour in relation to mental health and technology: implications for the development of online mental health services. BMC psychiatry 2013;13(1): 119.

## Appendix B. Factor loadings

| | |
|---|---|
| Can support therapist | 0.686 |
| Bridging distances | 0.743 |
| Bridging waiting time | 0.727 |
| Contemporary | 0.821 |
| Delivering evidence based treatment | 0.820 |
| Easy to share with family | 0.502 |
| Helping minorities / underserved | 0.731 |
| Improve self-management | 0.786 |
| Improvement of treatment quality | 0.832 |
| Independency from therapist | 0.630 |
| Low threshold to care | 0.777 |
| Online disinhibition effect | 0.752 |
| Psychoeducation | 0.732 |
| Repetition of work material | 0.672 |
| Suitable for generation 50+ | 0.765 |
| Suitable for young patients | 0.413 |
| Timewise flexible | 0.679 |
| Treatment intensification | 0.819 |
| Discrete | 0.553 |
| | |
| Risk of therapy discontinuation | -0.430 |
| Dealing with crisis | -0.602 |
| Data security issues | -0.442 |
| Too much technology | -0.809 |
| Not applicable for the majority | -0.703 |
| Lack of non-verbal signals | -0.773 |
| Avoidance of difficult situation | -0.745 |
| Missing problems in therap. Process | -0.754 |
| More complicated than classical therapy | -0.416 |
| Might result in side effects | -0.681 |
| Transfer into daily life | -0.732 |
| Missing important disease aspects | -0.753 |
| Technology devaluates therapist’s work | -0.678 |
